# No Evidence That Soluble TACI Induces Signalling via Membrane-Expressed BAFF and APRIL in Myeloid Cells

**DOI:** 10.1371/journal.pone.0061350

**Published:** 2013-04-19

**Authors:** Josquin Nys, Cristian R. Smulski, Aubry Tardivel, Laure Willen, Christine Kowalczyk, Olivier Donzé, Bertrand Huard, Henry Hess, Pascal Schneider

**Affiliations:** 1 Department of Biochemistry, University of Lausanne, Epalinges, Switzerland; 2 Adipogen SA, Epalinges, Switzerland; 3 Department of Patho-Immunology, Medical University Centre, Geneva, Switzerland; 4 Division of Hematology, Geneva University Hospital, Geneva, Switzerland; 5 Merck-Serono, Darmstadt, Germany; University of Miami, United States of America

## Abstract

Myeloid cells express the TNF family ligands BAFF/BLyS and APRIL, which exert their effects on B cells at different stages of differentiation via the receptors BAFFR, TACI (Transmembrane Activator and CAML-Interactor) and/or BCMA (B Cell Maturation Antigen). BAFF and APRIL are proteins expressed at the cell membrane, with both extracellular and intracellular domains. Therefore, receptor/ligand engagement may also result in signals in ligand-expressing cells via so-called “reverse signalling”. In order to understand how TACI-Fc (atacicept) technically may mediate immune stimulation instead of suppression, we investigated its potential to activate reverse signalling through BAFF and APRIL. BAFFR-Fc and TACI-Fc, but not Fn14-Fc, reproducibly stimulated the ERK and other signalling pathways in bone marrow-derived mouse macrophages. However, these effects were independent of BAFF or APRIL since the same activation profile was observed with BAFF- or APRIL-deficient cells. Instead, cell activation correlated with the presence of high molecular mass forms of BAFFR-Fc and TACI-Fc and was strongly impaired in macrophages deficient for Fc receptor gamma chain. Moreover, a TACI-Fc defective for Fc receptor binding elicited no detectable signal. Although these results do not formally rule out the existence of BAFF or APRIL reverse signalling (via pathways not tested in this study), they provide no evidence in support of reverse signalling and point to the importance of using appropriate specificity controls when working with Fc receptor-expressing myeloid cells.

## Introduction

TNF family ligands are type 2 membrane-bound proteins that form non-covalent trimers through an extracellular, carboxy-terminal domain of about 150 amino acid residues, coined the TNF homology domain [1]. BAFF (B cell Activating Factor of the TNF Family) is mainly expressed by myeloid cells and by radiation-resistant stromal cells [2], [3], [4]. It is synthesized as a membrane-bound protein that can be cleaved at a furin consensus sequence to release a soluble form of the cytokine. BAFF, but not APRIL (A PRoliferation-Inducing Ligand), stimulates B cell survival and controls the size of the mature B cell pool by engaging BAFFR expressed in transitional B cells and in naïve mature B cells (reviewed in [3]). BAFF and APRIL can also signal through TACI, a receptor whose expression is upregulated by Toll-like receptor signalling, and whose levels are particularly high in marginal zone B cells (reviewed in [5]). TACI^−/−^ mice have an enlarged B cell pool, indicating that TACI, unlike BAFFR, negatively regulates B cell numbers [6]. Despite having numerous B cells, TACI^−/−^ mice display strongly impaired T cell-independent type II antibody responses, in line with data showing that TACI engagement is required for survival of B cells activated by T-independent type II stimuli [6], [7]. BAFF and APRIL also promote plasma cell survival by engagement of BCMA, a receptor expressed during the latest B cell differentiation stages [8], [9]. We have previously shown that TACI stimulation in primary mouse B cells is inefficient using soluble trimeric BAFF or APRIL, but requires higher-order multimeric forms of the ligands that probably mimic the membrane-bound ligand [10]. Membrane-bound BAFF may thus be an important ligand for TACI, and conversely TACI may induce signalling in BAFF-expressing cells. Reverse-signalling has been described for cells expressing certain TNF family members [11], and in particular for BAFF and APRIL [12], [13], [14]. In the human monocyte cell line THP1, different anti-BAFF antibodies, but not a control mouse IgG antibody, induced, among others, phosphorylation of the mitogen-activated protein kinases ERK1/2, activation of the transcription factor NF-κB, secretion of the matrix metallo-protease 9 (MMP9), secretion of the chemokine IL-8 and upregulation of the adhesion molecule ICAM-1 [12]. IL-8 secretion was also observed in response to TACI-Fc but not human IgG. Similarly, anti-BAFF antibodies also increased, to some extent, MMP secretion in primary mouse macrophages [12]. It was concluded that BAFF-binding reagents trigger a (reverse) signalling event via membrane-expressed BAFF, leading to cellular activation [12]. Similar observations were made in THP1 cells stimulated with anti-APRIL antibodies [13]. Also, T-cell priming *in vivo* requires TACI-expressing B cells, and B cells can be replaced by TACI-Fc in this context [15].

BAFF is important for supporting B cell survival also in human, and administration of atacicept in patients reduces B lymphocyte numbers and immunoglobulin levels [16], [17]. Surprisingly, patients suffering from relapsing-remitting multiple sclerosis, after having been treated with atacicept, experienced exacerbation of disease as determined by some of the clinical endpoint measures. This fact resulted in the discontinuation of atacicept development in the context of central nervous system (CNS) inflammation [18].

In the present study, we investigated whether reverse signalling through membrane-expressed BAFF and/or APRIL can be detected in primary mouse cells in the presence of adequate controls, and whether this may provide a potential explanation for some of the effects of atacicept in CNS inflammation. We found that bone marrow-derived macrophages were indeed stimulated by TACI-Fc and BAFFR-Fc, but not by an irrelevant decoy receptor, Fn14-Fc, that target the TNF family ligand TWEAK. As confirmed in ligand-deficient cells, this activation was however independent of BAFF or APRIL expression, but dependent on the presence of protein aggregates that induced Fc receptor-mediated signalling.

## Results

### TACI-Fc and BAFFR-Fc, but not Fn14-Fc, Induce Signals in Primary Mouse Cells and Human Cell Lines

**Figure 1:**
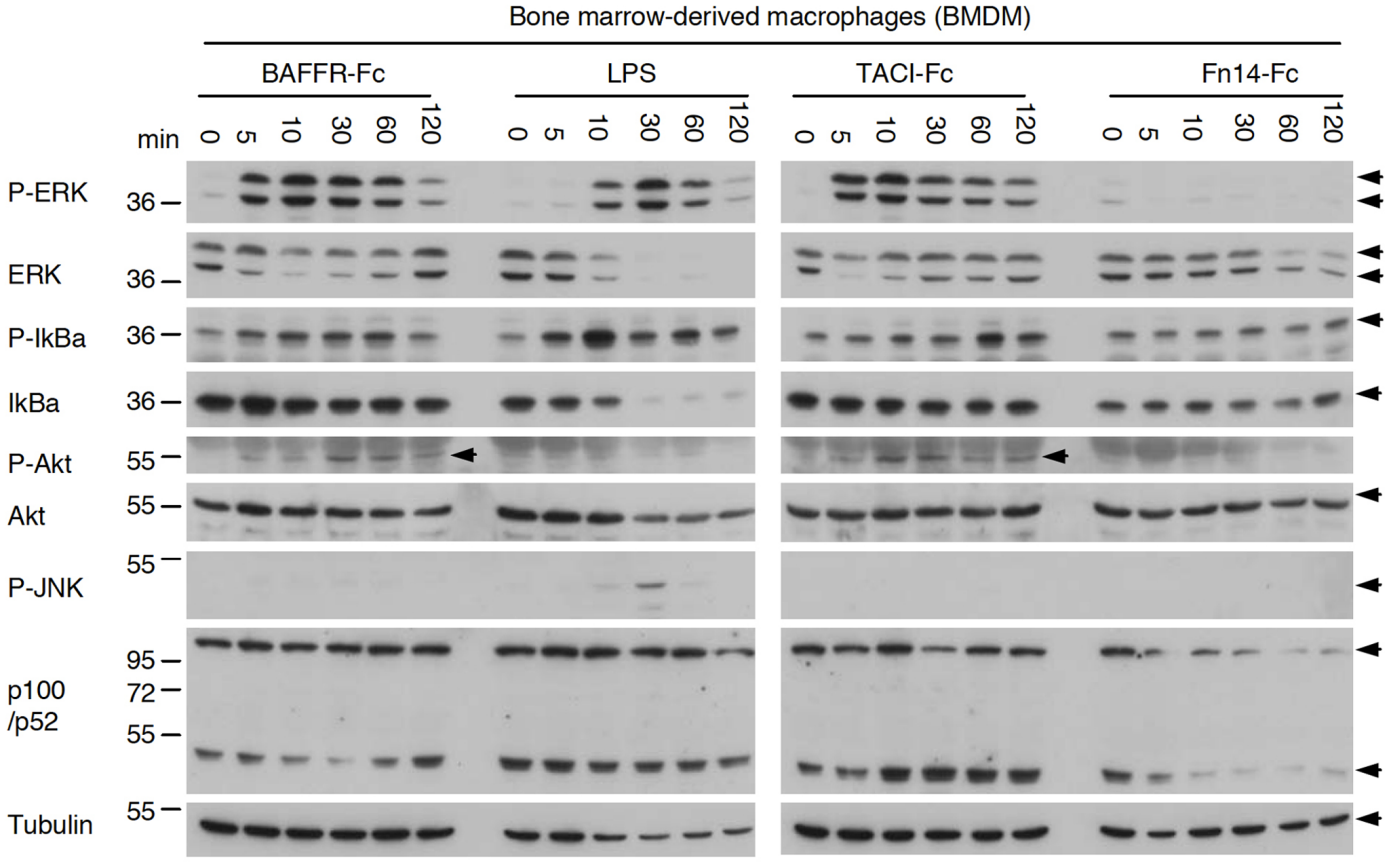
BAFFR-Fc and TACI-Fc, but not Fn14-Fc, induce signals in bone marrow-derived macrophages. Bone marrow-derived cells from C57BL/6 mice were stimulated for the indicated time with BAFFR-Fc, TACI-Fc or Fn14-Fc at 10 µg/ml, or with LPS at 50 ng/ml. Cells were lysed in sample buffer and analyzed by Western blotting with the indicated antibodies.

Primary mouse macrophages (BMDMs) exposed to endotoxin-free TACI-Fc or BAFFR-Fc displayed pronounced phosphorylation of ERK within 5 min of stimulation. In addition, Akt and IκBα were also phosphorylated, but neither TACI-Fc nor BAFFR-Fc appeared to significantly alter the p52 to p100 ratio, a hallmark of the alternative NF-κB pathway (Fig. 1A). None of these effects were observed with the negative control protein Fn14-Fc. Although stimulation of the same cells with LPS induced ERK and IκBα phosphorylation, this was achieved with kinetics different from those observed for TACI-Fc and BAFFR-Fc. LPS also induced JNK phosphorylation at higher levels than those obtained with TACI-Fc and BAFFR-Fc, further indicating that BAFFR-Fc and TACI-Fc effects were specific and not mediated by an endotoxin contamination (Fig. 1A). Similar but weaker activation signals were obtained in bone marrow-derived dendritic cells in response to TACI-Fc and, to a lesser extent BAFFR-Fc, but never with Fn14-Fc (data not shown).

**Figure 2:**
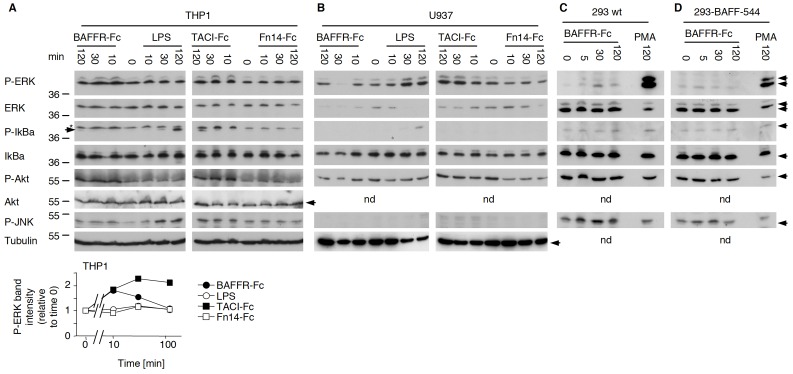
BAFFR-Fc and TACI-Fc, but not Fn14-Fc, induce signals in THP1 and U937 cells, but not in BAFF-expressing 293 cells. Panel A: THP1 cells were stimulated with BAFFR-Fc, TACI-Fc, Fn14-Fc (all at 5 µg/ml) or LPS (50 ng/ml) for the indicated times. In the P-IκBα blot, bands marked “*” are remnants of the P-ERK blot. The P-IκBα band is just underneath, indicated with an arrow. Bands in the P-ERK blot were quantified, normalized to the intensity of P-ERK at time zero, and plotted as a function of time. Panel B: same as panel A, except that the experiment was performed with U937 cells. Panel C: HEK-293 cells were stimulated with 5 µg/ml of BAFFR-Fc or with 100 nM PMA for the indicated time. Panel D: same as panel C, except that HEK-293 cells stably expressing full-length BAFF [22] were used. In all panels, all extracts were analyzed by western blotting using the indicated antibodies.

We next tested the human monocytic cell line THP1 and the human macrophage cell line U937 cells for their responses to BAFFR-Fc and TACI-Fc. Similar to what we observed in mouse macrophages, THP-1 cells responded to BAFFR-Fc and TACI-Fc, but not to Fn14-Fc, with increased ERK and Akt phosphorylation (Fig. 2A), and some ERK activation was also observed in U937 cells (Fig. 2B).

Taken together these results show that TACI-Fc and BAFFR-Fc, but not Fn14-Fc, can signal in myeloid cells of both mouse and human origin. Because of the Fn14-Fc control, we considered an Fc-mediated effect unlikely and instead favoured the hypothesis that TACI-Fc and BAFFR-Fc might exert their effects by inducing BAFF reverse signalling in these cells, all of which are known to express BAFF [19], [20], [21], [22].

### No Evidence for BAFF Reverse Signalling in 293 Cells Stably Expressing BAFF

Stimulation of HEK-293 cells stably expressing BAFF at their surface [22] with BAFFR-Fc induced no specific, BAFF-dependent ERK activation, and did not stimulate any of the other pathways examined (Fig. 2C and D). Stimulation with PMA and ionomycin could however activate ERK in these cells (Fig. 2C and D). Therefore, results obtained in macrophages could not be recapitulated in 293-BAFF cells, which is inconsistent with the BAFF reverse signalling hypothesis, unless HEK-293 cells would lack specific intermediates of the BAFF reverse signalling pathway.

### Signals Induced in Macrophages by BAFFR-Fc and TACI-Fc are Independent of BAFF and APRIL

**Figure 3:**
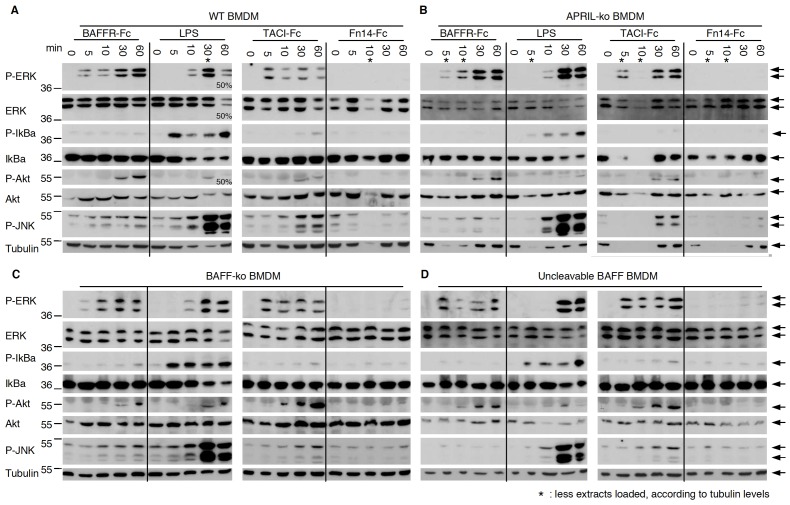
Signalling by BAFFR-Fc and TACI-Fc also takes place in APRIL^−/−^ and BAFF^−/−^ BMDMs, and is not increased in BMDMs with uncleavable BAFF. Bone marrow-derived macrophages from various mice in the C57BL/6 background were stimulated for the indicated times with BAFFR-Fc, TACI-Fc or Fn14-Fc at 5 µg/ml, or with LPS at 50 ng/ml. Cells were lysed and analyzed by Western blotting with the indicated antibodies. Panel A: wt BMDMs. Panel B: APRIL^−/−^ BMDMs. Panel C: BAFF^−/−^ BMDMs. Panel D: BMDMs from BAFF^−/−^ mice overexpressing non-cleavable BAFF from a BAC transgene [23]. *: as judged by the anti-tubulin blot, less sample was accidentally loaded.

We used a genetic approach to obtain definitive evidence as to whether or not membrane-expressed BAFF and/or APRIL are central mediators of a putative reverse signalling event as it has been observed in our experiments with primary macrophages. Macrophages derived from WT, APRIL^−/−^, BAFF^−/−^ mice or from mice expressing an excess of non-cleavable BAFF (fmBAFF-high Tg x BAFF^−/−^ mice [23]) were all activated in response to BAFFR-Fc and TACI-Fc, but not Fn14-Fc (Fig. 3). These results contradict the BAFF reverse signalling hypothesis that would have predicted increased signalling in cells expressing non-cleavable BAFF, reduced signalling in BAFF^−/−^ or APRIL^−/−^ cells stimulated with TACI-Fc and no signal at all upon stimulation of BAFF^−/−^ cells with BAFFR-Fc. Instead, results pointed to Fc-mediated effects, and we therefore searched to explain why our Fn14-Fc control failed to signal despite the presence of an intact Fc moiety.

### BAFFR-Fc, TACI-Fc and Fn14-Fc Differ in their Content of Polymeric Protein

**Figure 4:**
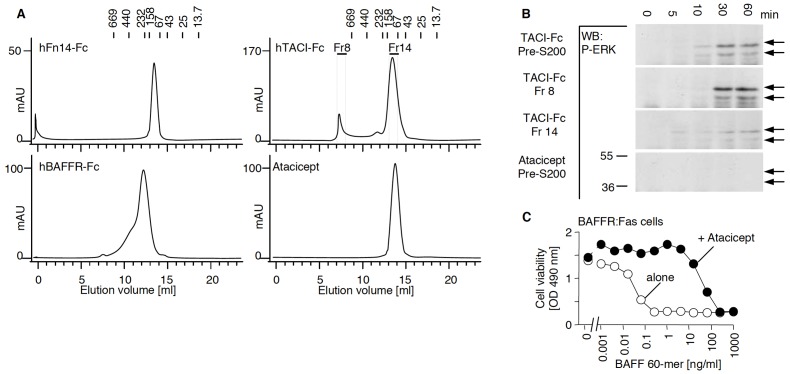
Gel filtration analysis of BAFFR-Fc, TACI-Fc, Fn14-Fc and atacicept. Panel A. Superdex-200 elution profiles of the indicated receptors-Fc monitored at 280 nm. The elution position of molecular mass standards is indicated at the top of the profiles. Panel B. BMDMs were treated with TACI-Fc obtained before (total preparation) or after size exclusion chromatography (fraction 8 with high molecular mass aggregates and fraction 14 with dimeric TACI-Fc). Cells were also treated with atacicept (before size fractionation), a form of TACI-Fc unable to bind to Fc receptors. Cell activation was monitored by western blotting with an anti-phospho ERK1/2 antibody. Panel C. BAFF-sensitive BAFFR:Fas expressing cells were stimulated with the indicated concentration of BAFF 60-mer, in the presence or absence of atacicept at a fixed concentration of 300 ng/ml. Cell viability was monitored with a colorimetric assay.

Gel permeation chromatography of Fn14-Fc showed that this protein was almost exclusively dimeric, whereas TACI-Fc displayed a significant proportion of high molecular mass aggregates. BAFFR-Fc presented an intermediate elution profile with a higher molecular mass shoulder in the chromatogram (Fig. 4A). Interestingly, the high molecular mass fraction of TACI-Fc readily induced ERK activation in primary macrophages, whereas the low molecular mass fraction displayed almost no such activity (Fig. 4B). We also tested atacicept, the good medical practice (GMP)-manufactured form of TACI-Fc used in clinical trials. The Fc portion of atacicept is engineered not to bind to Fc receptors or complement. Atacicept was exclusively dimeric by gel permeation chromatography and induced no ERK activation at all in mouse macrophages (Fig. 4A and B). We confirmed that atacicept used in this experiment was able to block BAFF using a reporter cell assay, in which the apoptotic Fas signalling pathway is triggered by BAFF in Fas sensitive cells via a BAFFR:Fas fusion receptor (Fig. 4C). Collectively, these data indicate that macrophage activation by TACI-Fc is dependent on multimers of TACI-Fc and is most likely mediated by engagement of Fc receptors, known to respond better to polymeric than to monomeric immunoglobulins.

### Signals Induced in Primary Mouse Macrophages by TACI-Fc is Fc-mediated

**Figure 5:**
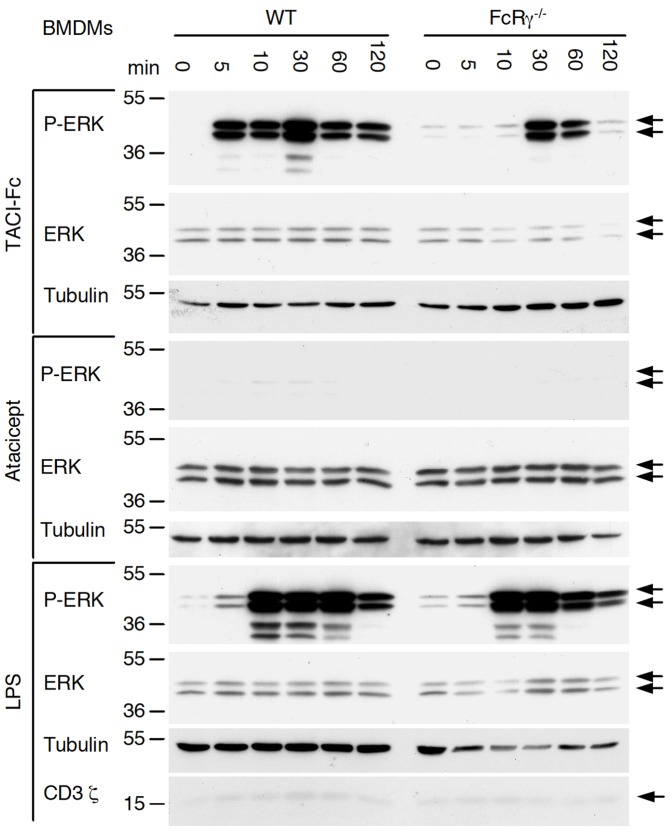
Impaired TACI-Fc signalling in FcRγ^−/−^ BMDMs. Bone marrow-derived macrophages from WT and FcRγ^−/−^ C57BL/6 mice were stimulated for the indicated times with TACI-Fc at 10 µg/ml, atacicept at 10 µg/ml, or with LPS at 50 ng/ml. Cell extracts were analyzed by western blotting with the indicated antibodies.

Several Fc receptors share a common γ chain, and FcRγ^−/−^ cells display impaired FcR signalling [24]. TACI-Fc-induced ERK activation was strongly reduced in macrophages deficient for FcRγ chain, especially after 5 or 15 min of stimulation. In contrast, stimulation with LPS resulted in robust ERK activation also in FcRγ^−/−^ cells (Fig. 5). It is noteworthy that TACI-Fc still induced some ERK activation in FcRγ^−/−^ cells after 30 or 60 min stimulation. This was probably Fc-mediated, because atacicept was totally inactive with respect to ERK activation (Fig. 5). Interestingly, an anti-CD3 ζ antibody detected a weak band of the expected size in BMDM extracts (Fig. 5). Taken together, these results indicate that ERK activation by TACI-Fc is mediated principally by Fc receptor(s) signalling via the γ chain, and accessorily by γ chain-independent effects, possibly via Fc receptors using the ζ chain.

## Discussion

It was shown previously that BAFFR-Fc and TACI-Fc have a tendency to form aggregated products that could in some cases be reduced by introduction of point mutations [25]. In this study, we showed that the agonist activity of TACI-Fc and BAFFR-Fc in different primary cells and cell lines was related to the presence of high molecular mass oligomers that formed the active fraction. These results could be explained by stimulation of Fc receptors on target cells rather than by BAFF-mediated reverse signalling. The hypothesis of BAFF reverse signalling was ruled out by the analysis of primary BMDMs, transgenic or knock-out for BAFF or APRIL, which were all stimulated as efficiently as wt cells. In particular, BAFF-deficient BMDMs responded to stimulation with hBAFFR-Fc which, unlike mBAFFR-Fc, does not interact with mouse APRIL at all [26]. The observed effect can be explained by stimulation of Fc receptors because i) receptor-Fc preparations devoid of oligomers (in this study atacicept and Fn14-Fc) did not induce activation, ii) activation still occurred in BAFF-deficient cells and iii) activation was impaired in FcRγ-deficient macrophages. Under natural conditions, cross-linking of FcR by immune complexes leads to activation of Scr family kinases that activate Syk family kinases. Numerous downstream effectors, including MAPK and NF-κB, are then activated [27], [28], that correspond to effects seen in our study. We have used U937 and THP1 cells because the former releases detectable levels of BAFF [19] and the latter was used to demonstrate BAFF-reverse signalling [12], [13], [14]. Of note, THP1 cells express FcRI and FcRII in a constitutive manner [29], and THP1 cells and U937 cells both respond to FcR stimulation by activating MAPK and NF-κB [28], [29]. In FcRγ^−/−^ macrophages, ERK activation in response to TACI-Fc was abolished at early time points. It was however still detectable at later time points. We do not believe that the residual signal was mediated via BAFF, APRIL or proteoglycans because atacicept that binds to BAFF, APRIL and proteoglycans but not FcR failed to stimulate macrophages. The residual signal obtained in FcRγ^−/−^ cells with TACI-Fc could be due to FcR signalling that is independent of the γ chain. For example, FcγRIII can also signal via the ζ chain. Although this was mainly described for NK cells [30], it may happen in BMDMs that apparently express the ζ chain. Paired immunoglobulin-like receptor A (PIR-A) that associates with the ITAM-containing β subunit of FcεRII, FcRn that can be active in myeloid cells, and binding of sialylated Fc to the C-type lectin SIGN-R1 expressed in macrophages may also explain Fc-mediated signals in BMDMs in the absence γ chain [31], [32], [33], [34]. It is probable that previously published papers, which lack adequate knock-out controls, wrongly interpreted FcR-mediated signals as BAFF – and possibly APRIL – reverse signalling [12], [13], [14].

Our experiments rule out an activation of ERK by BAFF reverse signalling in macrophages, but does not formally exclude that BAFF or APRIL reverse signalling may lead to activation of other targets that remain to be identified. Indeed, there is relatively solid evidence for reverse signalling by other TNF family members. For example, engagement of constitutively expressed OX40L on mast cells - by OX40-expressing regulatory T cells or OX40-positive cell membranes – inhibited immune complex-induced degranulation in WT cells, but not in OX40L-deficient cells or in cells blocked with an anti-OX40L monoclonal antibody [35]. It is also well documented that membrane-bound TNF or FasL are shed by cleavage with metalloproteases (TACE, ADAM10), leaving behind on the expressing cell the transmembrane segment and intracellular domain of the ligands. The transmembrane domain is thereafter processed by signal peptide peptidase-like aspartyl proteases that release in the producing cell the intracellular domains of TNF or FasL that can migrate and signal in the nucleus [36], [37], [38], [39]. The mechanism by which atacicept promoted CNS inflammation in patients with relapsing-remitting multiple sclerosis remains unknown. It could be related to a “true”, yet-to-be identified reverse signalling event, or to the ability of atacicept to interact with proteoglycans [40], or to unanticipated effects of plasma cell depletion, or to changes in the ratio between different B cell subsets. Indeed, BAFF-blocking agents preferentially deplete naïve B cells compared to switched memory B cells in human, the absolute number of circulating memory B cells being even increased during the first weeks of treatment [41], [42], [43], [44]. B cells contribute to the immune modulation of CNS inflammation [45], and in particular IL-10 production by B cells is important to control auto-immunity [46], [47], [48]. As BAFF can induce differentiation of marginal zone-derived IL10-producing B cells [49], its neutralization may weaken inhibitors of auto-immunity and exacerbate an already declared CNS inflammation.

## Methods

### Mice and Reagents

Mice were handled according to Swiss Federal Veterinary Office guidelines. Experiments with animals performed in this study were approved by the local institutional animal care and use committee and by the Office Vétérinaire Cantonal du Canton de Vaud (authorization 1370.3 to PS). C57BL/6 WT, BAFF^−/−^ and BAFF^−/−^ x fmBAFF-high mice [23], APRIL^−/−^ [50] and FcRγ^−/−^ [51] mice have been described before.

293 wt (ATCC CRL 1573) and 293-hBAFF full-544 cells were cultured in DMEM:F12 (1∶1 v/v) supplemented with 2% foetal calf serum [22] and THP1 (ATCC TIB-202) and U937 (ATCC CRL 1593) cells were cultured in RPMI supplemented with 10% foetal calf serum [19]. L929 mouse fibroblasts were cultured in RMPI supplemented with 10% FCS. Jurkat JOM2-BAFFR:Fas-2308 cl21 cells are Fas-negative Jurkat cells expressing a BAFFR:Fas fusion receptor. The BAFFR:Fas chimeric receptor comprises the following amino acid residues: MAIIYLILLFTAVRG (haemaglutinin signal peptide), LE, human BAFFR 2–71 (extracellular domain), EFGSVD, human Fas 169–355 (transmembrane and intracellular domains). These cells were generated as described for EDAR:Fas cells [52]. hTACI-Fc, hBAFFR-Fc and hFn14-Fc were from Adipogen. Atacicept was produced at Merck-Serono. Recombinant mouse GM-CSF was purchased from Immunoltools. *E. coli* K14 ultrapure LPS was from Invivogen. His-tagged BAFF 60-mer was from Adipogen. Antibodies for western blot were: mouse IgG1 anti-phospho-p42/44 (P-ERK) and mouse IgG1 anti-tubulin from Sigma, mouse IgG1 anti-phospho-IκBα, mouse IgG2b anti-phospho-Akt Ser473, rabbit anti-IκBα rabbit anti p100/p52, rabbit anti-Akt from Cell signalling, mouse IgG1 anti CD3 ζ and rabbit anti-pospho-JNK from Biosource and goat anti-ERK1 from Santa-Cruz.

### Bone Marrow-derived Macrophages (BMDM)

BMDMs were obtained as described [53]. Briefly, bone marrow of tibias and femurs was flushed in complete medium (RPMI, 10% FCS, 50 U/ml penicillin, 50 µg/ml streptomycin, 10 mM HEPES, 1 mM pyruvate, 50 µM 2-mercaptoethanol). Cells were harvested and incubated for 5 min at room temperature in 2 ml per leg of red blood cell lysis buffer (150 mM NH_4_Cl, 10 mM NaHCO_3_, 0.1 mM EDTA, pH7.3), washed in complete medium and seeded at 10^6^ cells/ml in BMDM differentiation medium (DMEM:F12, 10% FCS, 25% of L929 cell supernatant, 50 U/ml penicillin, 50 µg/ml streptomycin) in petri dishes for bacteriology. Cells were cultured at 37°C, 5% CO_2_ for 6 days, with addition on day 3 of 500 µl of fresh medium per ml of culture. Cells were then detached in PBS, 20 mM Hepes, 5 mM EDTA (or with Accutase solution, GE Healthcare), plated at 10^6^ cells/condition in 500 µl of RPMI 10% FCS in 48 well-plates, and left to attach overnight before stimulation.

### Cell Stimulation and Preparation of Cell Extracts

10^6^ BMDMs or BMDCs were stimulated for 0, 5, 10, 30, 60 or 120 min with various receptors-Fc at 5 µg/ml or with LPS at 50 ng/ml in 500 µl of RPMI 10% FCS. 3×10^5^ HEK-293 wt or HEK-293-BAFF cells were stimulated with hBAFFR-Fc at 5 µg/ml in 500 µl for the indicated times, or with 60 ng/ml of PMA. 4×10^4^ THP1 or U937 cells in 200 µl of RPMI 10% FCS in flat-bottom 96-well plates were stimulated for 0, 10, 30, 120 min with various receptors-Fc at 5 µg/ml or LPS at 50 ng/ml in 200 µl of RPMI 10% medium. At the end of the stimulation, medium was rapidly discarded and cells lysed immediately in 60 µl of 3 times concentrated, reducing SDS-PAGE sample buffer (30 µl for a well of a 96-well plate). Non adherent cells were first centrifuged for 5 min at 400×*g*. Extracts were transferred to 1.5 ml microtubes using wide-opening tips, heated for 5 min at 95°C and sonicated for 15 min in an ice-cold sonicating bath.

### SDS-PAGE and Western Blot

SDS-PAGE and Western blot were performed according to standard procedures, using the following number of cell equivalents per lane: 3×10^5^ for BMDM and BMDC, 10^5^ for HEK-293 cells and 4×10^4^ for THP1 and U937 cells. Western blots were revealed with the antibodies indicated in the figures, at dilutions indicated in Fig S1. In some cases, band intensity was monitored with the “Analyze gels” function of the ImageJ application (NIH).

### Gel Permeation Chromatography

Purified receptors-Fc were loaded in a volume of 400 µl on a Superdex-200 column (GE Healthcare) eluted in PBS with online absorbance monitoring at 280 nm and 1 ml fraction collection. Fractions of interest were filtered at 0.22 µm and concentration was calculated from absorbance at 280 nm using extinction coefficients of 1.328 for TACI-Fc and atacicept and 1.077 for BAFFR-Fc.

### Cytotoxicity Assay

The cytotoxicity assay using BAFFR:Fas cells was performed as described for FasL on Jurkat cells [54], except that graded amounts of BAFF 60-mer were used in the presence or absence of 300 ng/ml atacicept.

## Supporting Information

Figure S1 **List of antibodies used in this study.**
(PDF)
