# Supplementary material for: No Evidence That Soluble TACI Induces Signalling via Membrane-Expressed BAFF and APRIL in Myeloid Cells
Source: PLoS One. 2013 Apr 19;8(4):e61350. doi: 10.1371/journal.pone.0061350 (PMC3631189; doi:10.1371/journal.pone.0061350)
Supplement: Figure S1 — List of antibodies used in this study. (PDF) [file pone.0061350.s001.pdf]

Internal #	Target	Antibody		Supplier, Cat N°	Dil. WB
301	Phospho-p42/44 (P-ERK)	mIgG1	mAb	Sigma 015K4757	1/5000
303	Phospho-IκBα	mIgG1	mAb	Cell signalling 9246S	1/2000
306	Phospho-JNK	rabbit	pAb	Biosource 44-682 G	1/1000
322	Phospho-Akt Ser473	mIgG2b	mAb	Cell signalling 4051	1/1000
440	mIκBα	rabbit	pAb	Cell signalling 9242	1/1000
443	Tubulin	mIgG1	mAb	Sigma T5168	1/1000
556	ERK1	goat	pAb	Santa Cruz sc-094-G	1/2000
985	p100/52	rabbit	pAb	Cell signalling 4882	1/1000
1094	Akt	rabbit	mAb	Cell signalling 9272	1/1000
	CD3 zeta	mIgG1	mAb	Santa Cruz sc-1239	1/1000

Figure S1. **List of antibodies used in the study**.
Multiple revelations were performed in the following order

For Fig. 1: 301/985/322/556/1094 and 303/440/306/443.

For Fig. 2A: 332/306/301/303/440/443/556/1094.

For Fig. 2B: 322/306/303/301/440/556/443.

For Fig. 2C: 301/556/306 and 303/440/322.

For Fig. 3: 322/301/556/1094 and 306/303/440/443.
